# Fertility awareness and teenage pregnancy in rural western Uganda: a community-based cross-sectional study

**DOI:** 10.4314/ahs.v23i4.49

**Published:** 2023-12

**Authors:** Lilian Birungi, Jonathan Izudi

**Affiliations:** 1 Institute of Public Health and Management, Clarke International University, Kampala, Uganda. P.O. Box 7782, Kampala, Uganda; 2 Department of Community Health, Faculty of Medicine, Mbarara University of Science and Technology, Mbarara, Uganda. P.O. BOX 1410, Mbarara, Uganda; 3 Data Science and Evaluation Unit, African Population and Health Research Center, Nairobi, Kenya

**Keywords:** Teenage pregnancy, adolescent pregnancy, fertility awareness, Uganda

## Abstract

**Background:**

Teenage pregnancy (pregnancy among girls aged 13-19 years) is prevalent in Uganda but data about the associated factors are limited.

**Objective:**

To determine the prevalence and factors associated with teenage pregnancy in Buliisa district, western Uganda. We hypothesized that fertility awareness is associated with reduced likelihood of teenage pregnancy.

**Methods:**

In this cross-sectional study, we included girls aged 15-19 years and collected data using a structured questionnaire. The modified Poisson regression analysis was used to determine the association between fertility awareness and teenage pregnancy, adjusted for confounders, reported using adjusted prevalence risk ratio (aPR) and 95% confidence interval (CI).

**Results:**

Of 246 teenagers, the prevalence of teenage pregnancy was 41.5% while fertility awareness was 56.7%. The age category 18-19 years (aPR, 3.44; 95% CI, 2.16-5.47), fertility awareness (aPR, 1.80; 95%CI, 1.30-2.51) and Muslim faith (aPR, 1.37; 95%CI, 1.04-1.80) were associated with increased likelihood of teenage pregnancy. Living with a father (aPR, 0.61; 95%CI, 0.48-0.76), mother (aPR, 0.65; 95%CI, 0.524-0.81), or both parents (aPR, 0.57; 95% CI, 0.43-0.76) was associated with a reduced likelihood of teenage pregnancy.

**Conclusion:**

The prevalence of teenage pregnancy in Bulisa district is high. There is a need to target teenagers with correct fertility information, including the engagement of parents and religious leaders.

## Introduction

Teenage pregnancy, pregnancy among girls aged 13-19 years, is a devastating public health problem in low-income countries 1, with an estimated 17 million girls under the age of 19 giving birth every year and one million of them being under the age of 15 2. The majority of these pregnancies and childbirths are unplanned. Most low-income countries have high rates of teenage pregnancy 3. For instance, in sub-Saharan Africa, almost two in every 10 teenage girls are pregnant, with most of the pregnancies being in the East African region (21.5%) and the lowest in North Africa (9.2%) 4. Uganda has a teenage prevalence of 25% although there are differences between and within regions and districts 5.

Teenage pregnancy has numerous short and long-term effects. Teenage girls stand at a higher risk of giving birth to low-weight babies, developing pre-eclampsia or eclampsia, delivering before term (preterm delivery), and dying during delivery or the perinatal period compared to older women 6. There are equally several social complications, namely high rates of school dropout, unemployment, poverty, and domestic violence 2. Babies who are born to teenage mothers tend to die mostly during the perinatal days 7 and are more likely to be malnourished, have low mental and physical development, be deprived of social connectivity with their parents, and achieve worse educational outcomes 8 9. The high rates of death among teenage mothers also undermine efforts to reach the 2030 Sustainable Development Goal (SDG) target of reducing the global maternal mortality ratio to below 70 deaths per 100,000 live births 10.

Studies have shown that teenage pregnancy is high among rural adolescents, those who have ever married, and those whose father, mother, or both parents lack formal education 4. Sexual coercion, pressure from male partners, low or incorrect use of contraceptives, and inadequacies in parental communication and support have also been reported to increase the risk of teenage pregnancy on the African continent 11.

Observational studies have documented early marriage 12, peer influence 13, 14, viewing teenage girls as a source of wealth 15, 16, household poverty 17, limited access to and use of modern contraceptives 16, engagement in unprotected sexual intercourse 16, limited access to sex education, and lack of condom negotiation power 18 as factors that increase the risk of teenage pregnancy.

To understand how pregnancy occurs, every woman should know the time during her menstrual cycle within which she is most likely to conceive and every man needs to know that time 19. For women, being aware of such days of the month within which pregnancy is most likely (fertile days) can be used to achieve two goals. A woman can use this awareness to conceive by having sexual intercourse during her fertile days, or avoid pregnancy by not having sex, or using a condom or emergency contraceptive pills 20. Despite the benefits of fertility awareness in controlling pregnancy, its influence on teenage pregnancy has not been widely studied in most settings across sub-Saharan Africa.

The objective of this study was to determine the prevalence and factors associated with teenage pregnancy among girls aged 15-19 years in Buliisa district, western Uganda. We hypothesized that fertility awareness is associated with a reduced likelihood of teenage pregnancy. This evidence will inform the development of evidence-informed public health preventive strategies to lessen teenage pregnancy in the district and similar settings in sub-Saharan Africa.

## Methods and materials

### Study setting

We performed this study in Buliisa district in Western Uganda, a rural setting with a substantially higher prevalence of teenage pregnancy 21. Data show that 11.8% of teenage girls aged 15-19 years in the district have begun childbearing, 7.1% have given birth and 29.0% are pregnant with their first child 5. There are six sub-counties, one town council, 30 parishes, and 127 villages in the district. Buliisa district was curved out of Masindi district in July 2016 and lies in the western Albertine rift valley, a site known for its rich oil deposits. The district covers 3200 square kilometres and has 113,161 people, 50.2% of whom are below the age of 15, and just above half (51%) are females. There are 13,392 teenage girls, aged between 10 and 19 years, in the district of whom 10,647 have never been married 22. Buliisa district is predominantly rural and the majority of the population depends on peasant farming and fishing 23. Only 43% of the population is engaged in economic activities. Each household in the district has on average about six people, a little higher than the national average of four people per household. There are 11 functional health facilities in the district, namely one district hospital, one health centre IV (a sub-county level health facility), one health centre III (a parish level health facility), and eight health Centre IIs (village level) 5. About three in 10 people can access a government health facility within a 5 km radius of their residence 5.

### Study design, population, and sampling

We designed a community-based cross-sectional study that we conducted between November 07, 2019, and March 08, 2020. The study population consisted of girls aged 15-19 years since they are at risk for pregnancy. We adopted a multi-stage sampling approach since the distribution of the teenagers across the district was diverse. We randomly sampled three sub-counties, namely Buliisa, Butiaba, and Biiso as sites reporting teenage pregnancy. We then assigned unique identification codes to the parishes in the three selected sub-counties.

We used simple random sampling to select one parish from each sub-county, four villages from each parish, and then households within each village. The number of households per village depended on probability proportionate to the size of the village. The simple random sampling was performed using a computer-generated list of random numbers in R version 4.0.2 version after assigning unique codes at the sub-county, parish, and village levels. The selection of households was guided by unique identification numbers obtained from the local administrative unit. At the household level, eligible participants were consecutively sampled, one per household. For households with ≥2 eligible participants, simple random sampling was used, and for households without eligible participants, the nearest was selected.

### Measurements

The outcome variable was teenage pregnancy measured on a binary scale (no or yes). Participants were asked to state whether they had ever been pregnant or were pregnant at the time of the data collection. Participants who provided an affirmative response(s) to any of the two questions constituted teenage pregnancy. Teenage pregnancy was assigned the value of 1 otherwise 0. The prevalence of teenage pregnancy was computed as the proportion of participants aged 15-19 who reported ever being pregnant or were pregnant with their first child at the time of the study, expressed as a percentage. Fertility awareness was measured as a dichotomous variable (yes versus no). Participants were asked to state whether they were aware of their fertile days. Participants who provided affirmative responses were considered aware of their fertile days otherwise unaware. The potential confounders included age, religion, ethnicity, socio-economic status, confidence in making sexual decisions, receipt of advice from peers regarding sexual life, ability to negotiate safe sex, knowledge of emergency contraceptive use, knowledge of the importance of contraceptives, and access to contraceptives. Other variables included whether the participant was living with the mother, father, or both parents, and whether the parents had received formal education or not.

### Data collection and processing

Eight trained and supervised research assistants (two per village) collected the data using a pre-tested questionnaire following their recruitment through a local advertisement. The research assistants held at a minimum a diploma in health or social sciences, had previous experience in data collection, and were trained for two days on the study protocol, questioning techniques, and responsible conduct of research. The tool was pre-tested in Kigwera and Kihungya counties in Buliisa District on 10 teenagers per county. All the completed questionnaires were reviewed in real-time for completeness by a research Team Lead. Data were collected between 2.00 pm and 5.00 pm on Fridays, and between 9.00 am and 5.00 pm on Saturdays and Sundays to enable the participation of both in and out-of-school teenagers. Data were entered in Epi-Data version 3.1 along with data quality control measures, namely skip patterns, alerts, range, and legal values.

### Statistical analysis

We estimated that 282 participants were needed using OpenEpi 24, an online sample size calculator. We made the following assumptions: 1) 1050 teenagers aged 15-19 years in the three sub-counties 22; 2) 50% hypothesized frequency of teenage pregnancy; 3) 5% maximum allowable error; 4) 95% confidence level; and, 5) adjustment for a cluster design effect of 1. We summarized numerical data like age using the mean with the standard deviation when normally distributed otherwise the median with interquartile range (IQR) was used when skewed. We computed frequencies and percentages for categorical variables such as religion. We used the principal component analysis to compute socio-economic status (SES) based on the following assets: 1) material used for the construction of the roof, wall, and floor; 2) fuel used for cooking; 3) possession of a television set, radio cassette, bicycle, motorbike, mobile phone; 4) fuel used for lighting and, 5) type of toilet. These assets were selected to measure SES because they have been validated in Uganda and elsewhere 25.

In the analysis, the assets were divided into principal components and within each component, an asset with the highest factor loading after varimax rotation was selected. The analysis produced 11 components of which four were retained. We then selected an asset(s) with eigenvalues ≥1 under each of the retained components to compute the SES which we categorized as low, moderate, and high.

In the bivariate analysis, the Chi-square test was used to compare differences in teenage pregnancy with the categorical variables when the cell count was large (≥5), otherwise, the Fisher's exact test was used when the cell count was small (<5). To test mean differences in teenage pregnancy with numerical variables like age, we used the student's t-test as the data were normally distributed. The probability value (p-value) at the bivariate analysis was set at <0.15 to avoid residual confounding. In the multivariable analysis, we fitted a generalized linear model (GLM) with Poisson distribution, log-link, and robust standard errors to control for mild violations of the assumptions of Poisson regression 26, to determine the association between the independent variables and teenage pregnancy adjusted for potential confounders with p<0.15 at the bivariate analysis and from the literature. Our model considered both statistically significant and biologically plausible factors from the literature known to influence teenage pregnancy. We stated both the unadjusted and adjusted prevalence risk ratios (PR) together with the 95% confidence interval (CI). We preferred to use the modified Poisson regression to the binary logistic regression since the outcome was large. This is because, the odds ratio would overestimate the degree of association 27. We assessed the final model for possible multicollinearity using a variance inflation factor ≥10. The overall analysis was performed in R version 4.0.2.

### Ethical approval

This study received ethical approval from Clarke International University Research Ethics Committee (CIU-REC) and was assigned the reference number CIU-REC/0170). We obtained administrative letters of support from Buliisa District Health Office and the respective sub-county administrative offices. Participants aged 18 years gave written or thumb-printed informed consent after information had been provided on the study benefits and possible risks, purpose, and the right to withdraw or decline participation at any time. All the emancipated minors gave written informed consent although, for those below the age of 18 years, parental or the guardian's consent was sought in addition to assent.

### Reporting of findings

We adhered to the guidelines of the Strengthening of the Reporting of Observational Studies in Epidemiology (STROBE) 28 in reporting our results.

## Results

### Distribution of participant characteristics

**Figure 1:**
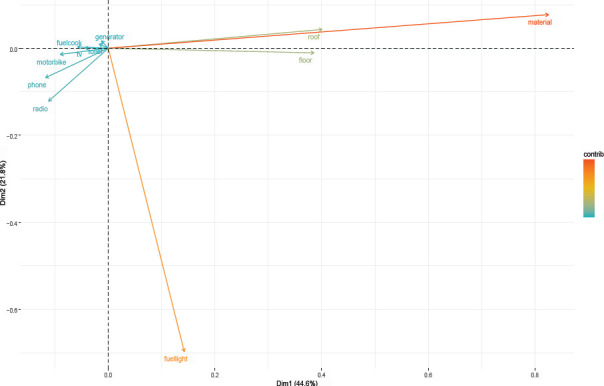
Eigenvalues graph showing distribution of assets

We studied 246 participants instead of 282 giving a response rate of 87.2%. The mean age of the participants was 17.3±1.5 years and 134 (54.5%) were in the age category of 18-19 years, 59 (24.0%) belonged to the Catholic religious faith, 139 (56.5%) belonged to families with low socioeconomic status, and 137 (55.7%) reported that they sometimes had confidence in making decisions about sexual health (Table 1). Figure 1 shows the eigenvalues graph and Table 2 shows the varimax rotation findings.

**Table 1:** Distribution of participant characteristics (n=246)

Characteristics	Level	n (%)
Age category in years	15-17	112 (45.5)
	18-19	134 (54.5)
	mean (SD)	17.3 (1.5)
Religion	Catholic	126 (51.2)
	Anglican	59 (24.0)
	Pentecostal	45 (18.3)
	Muslim	13 (5.3)
	Orthodox	3 (1.2)
Ethnicity	Bantu	80 (32.5)
	Non-Bantu	166 (67.5)
Socio-economic status	Low	139 (56.5)
	Moderate	44 (17.9)
	High	63 (25.6)
Confidence in making sexual health decisions	Never	50 (20.3)
	Most/all times	59 (24.0)
	Sometimes	137 (55.7)
Takes advice from friends or peers regarding sexual life	Never	37 (15.0)
	Most/all times	80 (32.5)
	Sometimes	129 (52.4)
Aware of fertile days	No	106 (43.1)
	Yes	140 (56.9)
Able to negotiate safe sex	Never/or no	69 (28.0)
	Most/all times	45 (18.3)
	Sometimes	132 (53.7)
Timing of emergency contraceptive use	After 72h	117 (47.6)
	Before 72h	129 (52.4)
Knows the importance of contraceptive	No	75 (30.5)
	Yes	171 (69.5)
Has access to contraceptives	No	56 (22.8)
	Yes	190 (77.2)
The person that the teenage lives with	None/ or alone	31 (12.6)
	Father only	49 (19.9)
	Mother only	75 (30.5)
	Both mother and father	91 (37.0)

**Table 2:** Varimax rotation table

Variables	Eigen values	Component	Mean
Material for construction of wall.	3.1	1	1.35
Material for construction of roof.	1.33	2	1.25
Material for construction of floor.	1.26	3	1.17
Fuel used for lighting.	1.06	4	1.11
Possession of generator.	1.00	5	1.05
Type of toilet.	0.85	6	0.99
Possession of a television (TV).	0.77	7	0.93
Fuel used for cooking.	0.73	8	0.88
Possession of a motorbike.	0.59	9	0.82
Possession of a phone.	0.19	10	0.76
Possession of a radio.	0.14	11	0.69

### Differences in teenage pregnancy with individual, parental, and socioeconomic factors at the bivariate analysis

In Table 3, our data show a teenage prevalence of 41.5% (102/246). Pregnant participants were on average older compared to those not pregnant: 18.3 (1.0) versus 16.62 (1.4), p<0.001. Teenage pregnancy was prevalent in the 18-19 years age category (86.3%), Catholic religious faith (51.0%), non-Bantu ethnicity (62.7%), participants from a low SES background (58.8%), and participants who sometimes made decisions regarding their sexual health (58.8%) among others. We observed statistically significant differences in teenage pregnancy concerning age, confidence in making decisions about sexual health, taking advice from friends/or peers regarding sexual life, fertility awareness, the timing of emergency contraceptive use, knowledge of the importance of contraceptives, and the person the teenager was living with at the time of the study.

**Table 3:** Differences in teenage pregnancy at bivariate level of analysis

Characteristics	Level	Teenage pregnancy		P value
		No (n=144)	Yes (n=102)	
Age category in years	15-17	98 (68.1)	14 (13.7)	<0.001
	18-19	46 (31.9)	88 (86.3)	
	mean (SD)	16.62 (1.4)	18.3 (1.0)	<0.001
Religion	Catholic	74 (51.4)	52 (51.0)	0.058
	Anglican	31 (21.5)	28 (27.5)	
	Pentecostal	33 (22.9)	12 (11.8)	
	Muslim	4 (2.8)	9 (8.8)	
	Orthodox	2 (1.4)	1 (1.0)	
Ethnicity	Bantu	42 (29.2)	38 (37.3)	0.232
	Non-Bantu	102 (70.8)	64 (62.7)	
Socio-economic status (SES)	Low	79 (54.9)	60 (58.8)	0.727
	Moderate	28 (19.4)	16 (15.7)	
	High	37 (25.7)	26 (25.5)	
Confidence in making sexual health decisions	Never	43 (29.9)	7 (6.9)	<0.001
	Most/all times	24 (16.7)	35 (34.3)	
	Sometimes	77 (53.5)	60 (58.8)	
Takes advice from friends or peers regarding sexual life	Never	29 (20.1)	8 (7.8)	0.004
	Most/all times	37 (25.7)	43 (42.2)	
	Sometimes	78 (54.2)	51 (50.0)	
Aware of fertile days	No	84 (58.3)	22 (21.6)	<0.001
	Yes	60 (41.7)	80 (78.4)	
Able to negotiate safe sex	Never/or no	45 (31.2)	24 (23.5)	0.253
	Most/all times	28 (19.4)	17 (16.7)	
	Sometimes	71 (49.3)	61 (59.8)	
Timing of emergency contraceptive use	After 72 hrs.	52 (36.1)	65 (63.7)	<0.001
	Before 72 hrs.	92 (63.9)	37 (36.3)	
Knows the importance of contraceptive	No	57 (39.6)	18 (17.6)	<0.001
	Yes	87 (60.4)	84 (82.4)	
Has access to contraceptives	No	35 (24.3)	21 (20.6)	0.596
	Yes	109 (75.7)	81 (79.4)	
The person the teenager lives with at the time of the study	None/ or alone	1 (0.7)	30 (29.4)	<0.001
	Father only	29 (20.1)	20 (19.6)	
	Mother only	47 (32.6)	28 (27.5)	
	Mother and father	67 (46.5)	24 (23.5)	
Parents received formal education	No	41 (28.5)	28 (27.5)	0.975
	Yes	103 (71.5)	74 (72.5)	

Note: SD: Standard deviation.

### Factors associated with teenage pregnancy at the unadjusted and adjusted analyses

In the unadjusted analysis (Table 4), our results show that participants aged 18-19 years compared to 15-17 years (unadjusted prevalence risk ratio (uPR),5.25; 95% CI, 3.44-8.03), those who belonged to the Muslim religious faith (uPR, 1.68; 95% CI, 1.18-2.38), had confidence in making decisions regarding sexual health at most/all times (uPR, 4.24; 95% CI, 2.32-7.75) or sometimes (uPR, 3.13; 95% CI, 1.72; 95% CI, 1.72-5.69), knew the importance of contraceptives (uPR, 2.05; 95% CI, 1.43-2.94), and took advice from friends or peers regarding sexual health at most or all times (uPR, 2.49; 95% CI, 1.45-4.28) had an increased likelihood of teenage pregnancy. However, teenage pregnancy was less likely among participants who had reported that emergency contraceptives are taken within 72 hours of unprotected sexual intercourse (uPR, 0.52; 95% CI, 0.40-0.67) and those who were living with their parents, namely the father (uPR, 0.42; 95% CI, 0.32-0.56), mother (uPR, 0.39; 95% CI, 0.30-0.50), or both (uPR, 0.27; 95% CI, 0.20-0.37).

**Table 4:** Factors associated with teenage pregnancy at the unadjusted and adjusted analyses

		Generalized linear model analyses	
Characteristics	Level	Unadjusted analysis, uPR (95% CI)	Adjusted analysis, aPR (95% CI)
Age categories (years)	15-17	1	1
	18-19	5.25**(3.44, 8.03)	3.44*** (2.16, 5.47)
Religion	Catholic	1	1
	Anglican	1.15 (0.86, 1.53)	0.76 (0.60, 0.98)
	Pentecostal	0.65 (0.42, 1.01)	0.79 (0.54, 1.15)
	Muslim	1.68* (1.18, 2.38)	1.37* (1.04, 1.80)
	Orthodox	0.81 (0.21, 3.13)	1.60 (0.66, 3.84)
Socio-economic status (SES)	Low	1	
	Moderate	0.84 (0.58, 1.21)	
	High	0.96 (0.71, 1.28)	
Confidence in making sexual health decisions	Never/none	1	1
	Most/all times	4.24***(2.32, 7.75]	1.42 (0.74, 2.72)
	Sometimes	3.13**(1.72, 5.69]	1.72 (0.94, 3.18)
Knows the importance of contraceptive	No	1	1
	Yes	2.05**(1.43, 2.94)	0.92 (0.63, 1.35)
Aware of fertile days	No	1	1
	Yes	2.75***(1.97, 3.85)	1.80** (1.30, 2.51)
Timing of emergency contraceptive use	After 72 hours	1	1
	Before 72 hrs	0.52***(0.40, 0.67)	0.88 (0.67, 1.15)
Takes advice from friends or peers regarding sexual health	Never	1	1
	Most/all times	2.49***(1.45, 4.28)	1.29 (0.77, 2.16)
	Sometimes	1.83* (1.06, 3.15)	1.06 (0.64, 1.74)
The person that the teenager lives with at the time of the study	None/ or alone	1	1
	Father only	0.42***(0.32, 0.56)	0.61**(0.48, 0.79)
	Mother only	0.39***(0.30, 0.50)	0.65**(0.52, 0.81)
	Both mother and father	0.27***(0.20, 0.37)	0.57***(0.43, 0.76)
Parents received formal education	No	1	
	Yes	1.03 (0.78, 1.36)	

Note: 1); * p < 0.05; **p < 0.001; *** p<0.001 at 5% significance level; 2) Prevalence risk ratios (PR) are exponentiated with confidence intervals in brackets; 3) aPR: Adjusted prevalence risk ratio; 4) uPR: Unadjusted prevalence risk ratio.

In the adjusted analysis (Table 4), the factors associated with a more likelihood of teenage pregnancy included the 18-19 years age category compared to 15-17 years (adjusted prevalence risk ratio (aPR), 3.44; 95% CI, 2.16-5.47), belonging to the Muslim religious faith (aPR, 1.37; 95% CI, 1.04-1.80) and being aware of fertile days (Adjusted PR (aPR), 1.80; 95% CI, 1.30-2.51). However, teenage pregnancy was less likely among participant who were living with their parents, namely the father (aPR, 0.61; 95% CI, 0.48-0.79), mother (aPR, 0.65; 95% CI, 0.52-0.81), or both (aPR, 0.57; 95% CI, 0.43-0.76).

## Discussion

The focus of this study is on the prevalence and factors associated with teenage pregnancy in Buliisa district in rural western Uganda. Our data show that more than half of the teenagers are aware of their fertile days and at least four in ten are either pregnant or have ever been pregnant. We found fertility awareness is associated with a nearly two-fold risk of teenage pregnancy. The other factors associated with an increased risk of teenage pregnancy include increasing age and being a Muslim while living with a father, mother, or both parents is associated with reduced risk of teenage pregnancy. The prevalence of teenage pregnancy in this study is relatively higher than the national average of 25% 5 and that for sub-Saharan Africa at 19.3% 4 and this might exacerbate maternal and newborn morbidity and mortality as teenage pregnancy is associated with numerous complications, namely obstructed labor, pre-term delivery, low birth weight among others 6. The level of fertility awareness is consistent with the findings of a previous study in Uganda that found low levels of fertility awareness among female university students 19. Another study conducted in Kenya found high levels of incorrect information about menstruation and fertility, with most participants not knowing the correct times when pregnancy could occur 29. Therefore, our results underscore the need for family planning counsellors or service providers and parents to provide age-appropriate fertility and sexual and reproductive health information to teenage girls.

The finding that fertility awareness is associated with an increased likelihood of teenage pregnancy requires cautious interpretation since we did not establish the source of sexual and reproductive health information. First, fertility awareness can be used to prevent unplanned pregnancy by avoiding sex during the fertile days or to achieve pregnancy by engaging in unprotected sexual intercourse 30. Although fertility awareness is considered as being safe, cheap, user-dependent, and acceptable to some religions as opposed to hormonal contraceptives, it does not protect against sexually transmitted infections (STIs) including HIV. Furthermore, fertility awareness is limited by its lack of effectiveness in preventing unwanted pregnancies, with an estimated 30 pregnancies per 100 women per year 30, 31. The method is not appropriate for women with irregular menstrual periods as this makes it hard to differentiate between safe and fertile days. Accordingly, the increased likelihood of teenage pregnancy could be due to these limitations. Another socially plausible explanation could be incorrect information about fertile days. In Nepal, peers are the most common source of sexual and reproductive health information for adolescents, and most of the information they received was inaccurate leading to an increased likelihood of teenage pregnancy 32. Our finding might be explained by the inaccuracy of information about fertile days as was the case in Nepal. A previous study reports that the majority of adolescents receive information about menstruation and fertility through schools, non-governmental organizations (NGOs), family members, or friends 29. However, the information they recieve lacks accuracy 29. For example, teenage girls were told that they could become pregnant once they had started to menstruate, which is incorrect. This is consistent with the findings of this study since the majority of participants who sometimes or at all times took advice from friends ended up becoming pregnant, suggesting that the sources of information about menstruation and fertility are questionable. Accordingly, there is a need for providing correct information regarding menstruation and fertility among adolescents. Lastly, assuming that the participants knew their fertile days, then, the pregnancy could have been intended. Studies conducted in other settings have reported intended pregnancy as a growing public health problem among adolescents 16, 33.

Our study shows that the likelihood of teenage pregnancy is higher among those aged 18-19 years compared to 15-17 years, which is not surprising since sexual activity increases as teenagers mature. Adolescents , therefore, tend to engage in sexual relationships with their peers as well as older men (age-disparate relationship), with the latter characterized by condomless sexual intercourse 34. Our finding is consistent with a previous study that reported a high likelihood of teenage pregnancy with increasing age 35. Previous studies report that the majority of girls within the 15–19-year age group do not carry or even buy condoms from health facilities due to stigma and fear of being reported to their parents or caregivers by health workers 3, 32.

Therefore, fear compounded by stigma might have contributed to the increased likelihood of pregnancy. This result implies that age-appropriate sexual and reproductive health services should be made readily available and accessible for teenage girls, and the services must be provided in a confidential and non-judgmental manner.

Our finding of a reduced likelihood of teenage pregnancy among participants who live with either one or both parents is consistent with a previous study which reports a lower likelihood of teenage pregnancy when parents are available 2, possibly due to parental guidance. Parenting is associated with better development of sexual and reproductive health 36, largely because parents provide information about sexual and reproductive health matters including the prevention of human immunodeficiency virus (HIV) infection 37. Indeed, poor parental communication and support increase the likelihood of teenage pregnancies 11. When parents live together with their children, a good, positive, and strong relationship are established and this potentially contributes to the right moral values 36.

We found that the Muslim religious faith is associated with a higher likelihood of teenage pregnancy. Early childbearing is a common occurrence among most Muslim families and a sensitive issue to discuss because it is shaped by religious teachings. Although Islamic teachings promote the care of pregnant mothers and babies, it is reported that the teachings are not well understood or even applied to Muslim communities 38. One study report that teenage pregnancy among Muslim girls is a result of the failure of parents to control their daughter's behaviour and the existence of communication problems between parents and teenagers 39.

## Study strengths and limitations

This study has some strengths and limitations. Our study is the first to report on the association between fertility awareness and teenage pregnancy. However, our study did not establish the kinds of sexual and reproductive health information discussed between parents and teenage girls and future studies should seek to underscore this limitation. We did not account for the variation of teenage pregnancy at village and sub-county levels since multi-level modeling was not performed. We acknowledge that there is a possibility of social desirability bias and that the findings might not be generalizable to urban settings due to differences in socio-economic profiles between rural and urban teenage girls. The lack of a pregnancy test could have resulted in an underestimation of the study outcome. The use of multi-stage sampling potentially increases sampling bias. These limitations should be considered in the interpretation of the results.

## Conclusions and recommendations

Our study shows a high prevalence of teenage pregnancy in Buliisa district, western Uganda. We found fertility awareness is associated with a higher likelihood of teenage pregnancy as well as the age category 18-19 years and belonging to the Muslim religious faith. Teenage girls who live with either one or both parents have a reduced likelihood of pregnancy. We recommend that these factors should be the focus for designing sexual and reproductive health interventions and messages for the prevention of teenage pregnancy in the district and similar settings in Uganda and elsewhere. In particular, there is a need to target teenagers with correct information about fertility, including the engagement of parents and religious leaders in tackling teenage pregnancy.
